# Corticotropin releasing factor-binding protein (CRF-BP) as a potential new therapeutic target in Alzheimer’s disease and stress disorders

**DOI:** 10.1038/s41398-019-0581-8

**Published:** 2019-10-22

**Authors:** Dorien Vandael, Natalia V. Gounko

**Affiliations:** 1 VIB-KU Leuven Center for Brain and Disease Research, Electron Microscopy Platform, Herestraat 49, B-3000 Leuven, Belgium; 2 VIB Bioimaging Core Facility, Herestraat 49, B-3000 Leuven, Belgium; 3 KU Leuven Department of Neurosciences, Leuven Brain Institute, Herestraat 49, B-3000 Leuven, Belgium

**Keywords:** Molecular neuroscience, Pharmacology

## Abstract

Alzheimer’s disease is the most common cause of dementia and one of the most complex human neurodegenerative diseases. Numerous studies have demonstrated a critical role of the environment in the pathogenesis and pathophysiology of the disease, where daily life stress plays an important role. A lot of epigenetic studies have led to the conclusion that chronic stress and stress-related disorders play an important part in the onset of neurodegenerative disorders, and an enormous amount of research yielded valuable discoveries but has so far not led to the development of effective treatment strategies for Alzheimer’s disease. Corticotropin-releasing factor (CRF) is one of the major hormones and at the same time a neuropeptide acting in stress response. Deregulation of protein levels of CRF is involved in the pathogenesis of Alzheimer’s disease, but little is known about the precise roles of CRF and its binding protein, CRF-BP, in neurodegenerative diseases. In this review, we summarize the key evidence for and against the involvement of stress-associated modulation of the CRF system in the pathogenesis of Alzheimer’s disease and discuss how recent findings could lead to new potential treatment possibilities in Alzheimer’s disease by using CRF-BP as a therapeutic target.

## Release of CRF in response to stress

Stress is a life-saving mechanism that has been shaped and refined throughout evolution^1^. Acute stress leads to an increase in attention and to memory consolidation^2,3^. For example, anxiety is a normal reaction to stress and, if not excessive, is crucial for homeostasis. However, chronic or excessive stress leads to a decrease in performance and cognition, and hence limited adaptation to the stressor^4,5^. Individual kinetics and magnitude of stress response determine its outcome in terms of resilience or development of stress-related disorders^6^. This can occur in every stage of life^5,7^. Already before birth, stressful situations can have a major impact on the future life span of the organism as well as in newborn, young adult, and older stages of life^7,8^.

**Fig. 1:**
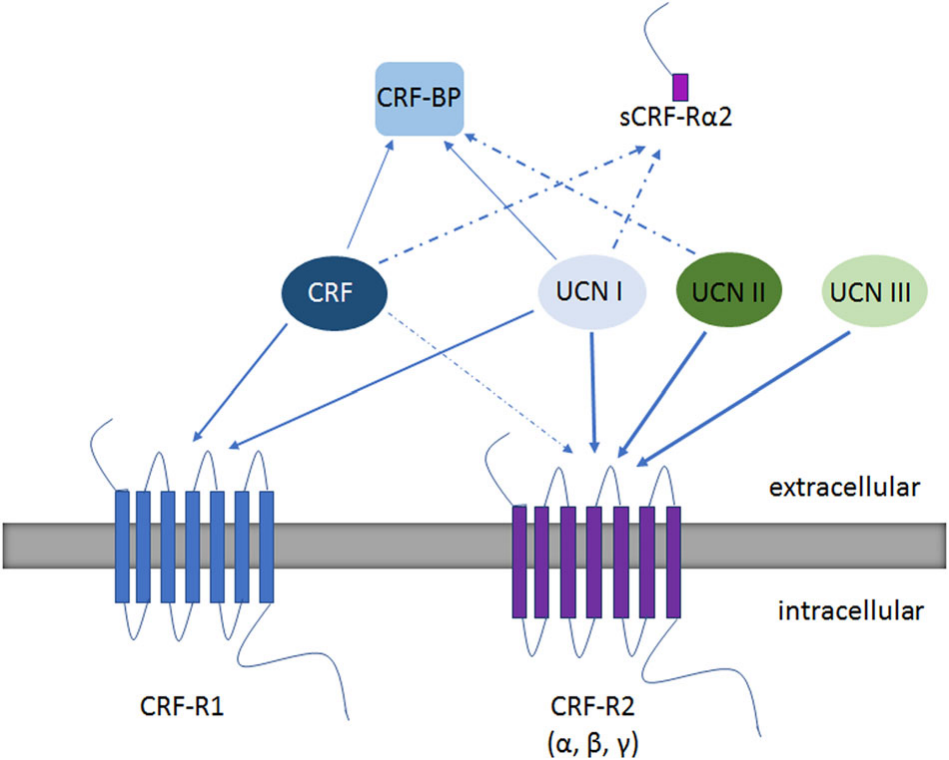
The CRF family and its relation with CRF-BP. The CRF family consists of two different CRF receptors: CRF-R1 (one functional isoform) and CRF-R2 (three functional isoforms: *α*, *β*, *γ*). The ligands, CRF and UCN I, UCN II and UCN III will bind and will induce G-protein-coupled signaling via CRF-Rs. The arrows represent the affinity between ligand and receptor or ligand and binding protein. The different affinities are represented by the pattern and thickness of the arrow lines; dashed lines will represent lower affinity as compared with solid arrow lines. The weight of the solid lines will give even more detail about the affinity between both ligand and receptor. CRF displays a relatively high affinity for CRF-R1 and CRF-R2. CRF has a comparatively lower affinity for CRF-R2 compared with its affinity for CRF-R1. UCN I has an approximately equal affinity for both receptors, and UCN II and UCN III seem to be selective for CRF-R2. The signaling cascade also includes CRF-BP and the recently identified soluble α-isoform of CRF-R2 (sCRF-Rα2). CRF-BP binds to CRF and UCN I with high affinity to modulate the biological activities of the ligands. Both CRF-BP and CRF-Rα2 are able to sequester CRF and UCN I, whereas CRF-BP exerts a low affinity for UCN II. Abbreviations; corticotropin-releasing factor (CRF), urocortin (UCN), CRF receptor 1 (CRF-R1), CRF receptor 2 (CRF-R2), CRF-binding protein (CRF-BP), α-soluble isoform of CRF-R2 (sCRF-Rα2)

Stress response is a highly orchestrated mechanism whereby the body rapidly activates the autonomic nervous system and the hypothalamic-pituitary-adrenocortical (HPA) axis^9,10^. By activation of the HPA axis and the autonomic nervous system, an enormous number of hormones, neurotransmitters, and neuropeptides are released as adaptive reactions to restore homeostasis^10^. Corticotropin-releasing factor (CRF) and CRF family peptides (Fig. 1) are major regulators of stress response due to their ability to integrate physiological responses to react against a stressor, and due to their dual roles as hormones and as neuromodulators^10–12^.

As a hormone, the 41-amino acid polypeptide CRF is secreted at the onset of stress in the paraventricular nucleus of the hypothalamus^13^. CRF is delivered via the bloodstream to the anterior pituitary, where it binds to its receptors and stimulates adrenocorticotropic hormone (ACTH) release. ACTH release activates synthesis of corticosteroids in the adrenal cortex: glucocorticoids such as cortisol in humans and corticosterone in rodents^13,14^. Glucocorticoids can exert profound modulatory effects on a variety of brain functions from early sensitive developmental stages to late adulthood. Fetal exposure to exogenous glucocorticoids or prenatal stress can lead to permanent alteration of HPA function and stress-related performance^15^. At the adult stage, high levels of glucocorticoids have been associated with reduced cognitive ability, including poor memory and decreased mental flexibility and processing speed^16^. After crossing the blood–brain barrier, glucocorticoids activate two types of receptors, the glucocorticoid receptor (GR) and the mineralocorticoid receptor (MR), which mediate stress response in the brain^6,17^. MRs are active under basal conditions and have high affinity to glucocorticoids. GRs have low affinity to glucocorticoids and are activated in response to high levels of the hormone during stress^18,19^. To summarize, endogenous corticosteroid secretion from the adrenal cortex is mainly under the control of ACTH produced by the pituitary gland. ACTH secretion is controlled mostly by the hypothalamic CRF. The aforementioned phenomena rely on transcriptional regulation and occur within time frames ranging from hours to weeks^20^.

## Roles of CRF as a neuromodulator

In contrast to hormones, neurotransmitters and neuromodulators exhibit fast action, allowing neurons to react to stressful situations on the synaptic level, within milliseconds to minutes^20^. CRF acts as the principal hormone in the HPA axis^21^, as discussed above, and triggers a number of secondary stress-related events such as secretion of the corticosteroids in the adrenal cortex. However, in addition to its action to regulate the HPA axis at the level of the pituitary, CRF is also expressed in different parts of the brain, where it can act as a neuromodulator or neurotransmitter in response to autonomic and behavioral responses^22^. Here, CRF synergizes with corticosteroids to fine-tune stress responses in a short time frame^11,23,24^.

CRF binds to two receptors, CRF-R1 and CRF-R2, with a higher affinity for CRF-R1^23^ (Fig. 1). Both are seven-span G-coupled receptors, are expressed in different regions in the brain, and share ~70% identity with each other^25–28^. In addition to CRF, these receptors can bind three other ligands (Fig. 1): urocortin (UCN) I, UCN II (stresscopin-related peptide), and UCN III (stresscopin). UCN I can bind to both receptors with similar affinity^29^, UCN II shows preferential binding to CRF-R2, and UCN III selectively binds CRF-R2^23^. Binding of CRF and UCN I to CRF-R1 and CRF-R2 is modulated in vivo and in vitro by the CRF-binding protein (CRF-BP)^30^, a 37 kDa glycoprotein that was first detected in human plasma^31–33^. CRF-BP is structurally unrelated to CRF receptors^34^. In vertebrates, CRF-BP binds to both CRF (Ki = 0.2 nM) and UCN I (Ki = 2 nM) with sub-nanomolar affinity (Fig. 1 and Table 1), which is greater than the affinity between CRF and its receptors. The affinity of CRF-BP for the other CRF family members is substantially lower: for UCN II, the IC50 is 4.4 nM and UCN III has no affinity for CRF-BP, only for CRF-R2^30,35^. This suggests that CRF-BP could differentially modulate the activity of several CRF family members.

**Table 1:** A summary of the most common agonists and antagonists of the CRF family

	Agonist					Antagonist				
	Name	Peptide/nonpeptide	Peptide sequence	Binding affinity Ki (nM)	References	Name	Peptide/nonpeptide	Peptide sequence	Binding affinity Ki (nM)	References
CRF-R1	h/r CRF	Peptide	SEEPP**ISLDL**T FHLLREVLEM ARAEQLAQQA HSNRKLMEII	1.0 (0.2–4.6)	^109, 131^	Alpha-helical CRF fragment 9–41	Peptide	DLT FHLLREMLEM AKAEQEAEQA ALNRLLLEEA	19 (5.5–66)	^108, 129, 130^
						Astressin	Peptide	fHLLREVLEZ ARAEQLAQEA HKNRKLZEII	0.7 (0.3–1.8)	^101, 129^
						Antalarmin	Non-peptide	NA	1	^110, 132^
						CP-154,526	Non-peptide	NA	2.7	^110, 132^
						CP-316,311	Non-peptide	NA	6.8	^110, 111^
	h UCN I	Peptide	DNPS**LSIDL**T FHLLRTLLEL ARTQSQRERA EQNRIIFDSV	0.1 (0.1–0.2)	^109, 131^	CRA-0450	Non-peptide	NA	40–60	^110, 133^
						NBI-30775/R121919	Non-peptide	NA	3.5	^114^^,^^134^^,^^135^
						Pexacerfont	Non-peptide	NA	7.2 ± 0.9	^113^ ^, 136^
						Verucerfont	Non-peptide	NA	6.1	^136^
CRF-R2	h/r CRF	Peptide	SEEPP**ISLDL**T FHLLREVLEM ARAEQLAQQA HSNRKLMEII	6.2 (2.0–19)	^109, 131^	Antisauvagine 30	Peptide	fHLLRKMIEI EKQEKEKQQA ANNRLLLDTI	1.4	^130^
	h UCN I	Peptide	DNPS**LSIDL**T FHLLRTLLEL ARTQSQRERA EQNRIIFDSV	0.5 (0.3–0.7)	^109, 131^					
	h UCN II	Peptide	IV**LSLDV** IGLLQILLEQ ARARAAREQA TTNARILARV	0.5 (0.2–1.2)	^109, 131^	Astressin_2_-B	Peptide	DLS FHLLRKXIEI EKQEKEKQQA ENNKLLLDLI	1.3	^130^
	h UCN III	Peptide	FT**LSLDV** TNIMNLLFNI AKAKNLRAQA AANAHLMAQI	13.5 (9.2–19.7)	^109, 131^					
CRF-BP	CRF	Peptide	SEEPPISLDLT FHLLREVLEM ARAEQLAQQA HSNRKLMEII	0.2	^109, 131^	h/r CRF_6-33_	Peptide	ISLDLTFH LLREVLEMAR AEQLAQQAHS	0.2–5	^40^
	UCN I	Peptide	DNPSLSIDLT FHLLRTLLEL ARTQSQRERA EQNRIIFDSV	2	^109, 131^	h/r CRF_9-33_	Peptide	DLTFH LLREVLEMAR AEQLAQQAHS	0.2–5	^40^

In bold, receptor activation; underlined, ECD1-binding domain; blue letter, CRF-R2 specific; double underlined, CRF-BP sequence; Ki, binding affinity

The discovery of a rodent-specific splice variant α-isoform of CRF-R2 (sCRF-Rα2), which encodes the soluble ligand-binding domain of CRF-R2^36^, suggests an even greater complexity of the CRF family. Similar to CRF-BP, sCRF-Rα2 binds CRF and UCN I but has a distinct distribution in comparison with CRF-BP^36,37^ (Fig. 1). sCRF-Rα2 is highly expressed in the olfactory bulb, cortex, midbrain, and the pituitary, and lower levels are found in the hypothalamus, pons, medulla, and spinal cord, and shows high overlap with the cellular distribution of CRF-R1^36,38^. In the CNS, CRF-BP is highly expressed in the cortex, olfactory bulb, hippocampus, and amygdala, and is known to inhibit CRF actions in the HPA axis^39,40^. In the brain, CRF-BP function depends on the tissue and cellular context^41^. Here, CRF-BP and CRF exhibit limited co-expression at the cellular level^36,38^. CRF-BP is localized at the CRF target sites such as the prefrontal cortex and amygdala, regions important in stress-related conditions including anxiety and addiction^41^. Notably, there is a difference in expression of the CRF-BP between male and female mice. In females, CRF-BP expression is positively regulated by estrogen and gonadotropin-releasing hormone in pituitary cell types. This increased expression positively correlates with several conditions such as anorexia, mood disorders, and anxiety disorders, which are more prevalent in females^42^.

In addition to its functions in stress, CRF exerts time- and dose-dependent direct actions during learning and memory formation. The exposure of neurons to acute CRF in vitro induces adaptation by spine increase and spine maturation, but exposure to high dosages of the peptide results in spine retraction and loss of synapses in the hippocampus^20,43^. Opposite effects were seen in the cerebellum, where an increase of spines and dendritic complexity was observed in response to physiological concentrations of CRF in organotypic slice cultures^44,45^. Clearly, effects of CRF on cellular plasticity and connectivity are highly dependent on the context, i.e., the administered dose, duration of stimulation, and cellular composition. It was further shown that chronic exposure or high levels of CRF lead to the activation of CRF-R1, and subsequently actin cytoskeleton rearrangements and atrophy of the spines and dendrites over longer time periods^46,47^. This decrease in spines and in dendritic complexity, and changes in synaptic function are cellular hallmarks of cognitive impairment^20^, characteristic, among others, for late stages of neurodegenerative disorders such as Alzheimer’s disease^48–50^.

## Stress as an environmental risk factor in Alzheimer’s disease

Alzheimer’s disease (AD) is an age-dependent neurodegenerative disorder that causes progressive cognitive impairment and structural alterations in the brain. The brains of patients with AD are characterized by the deposition of extracellular amyloid plaques consisting of β-amyloid (Aβ) peptides, the product of the proteolytic processing of the amyloid precursor protein (APP), and by abnormal hyperphosphorylation of the Tau protein, which precipitates in the form of neurofibrillary tangles inside the neurons^48,49^. A hallmark of the disease is the progressive neuronal loss in the hippocampus and cortex, brain structures that are extremely important in memory formation^50–52^. The most common form of AD is sporadic AD without a familial link, but genetically the disease presents as both familial and sporadic cases^53^. Despite many years of research, whether all forms of AD have a common underlying etiology remains poorly understood. A major effort in the research field has been put into the investigation of genetic factors, although environmental factors could be similarly or even more important in the onset of the disease^54^.

For example, in an AD mouse model, carrying a mutated form of APP, accumulation of Aβ and hyperphosphorylated Tau has been shown to occur as a result of exposure to high stress levels by acute restrained stress, chronic isolation, and social stress paradigms^55–57^. A reduction in the number of mature dendritic spines in a triple-transgenic mouse model of AD (3 × Tg-AD: PS1 (M146V), APP (Swe), tau (P301L)) and increased Aβ levels due to the augmentation of amyloid-β protein precursor processing^58^ have been observed in response to short-term stress.

Exposure to stress early in life can contribute to the later development of AD^59–62^. High and chronic stress levels lead to a hyper-activation of the HPA axis, which correlates with the rate of AD progression^63,64^. At the same time, chronic stress and stress-dependent disorders, such as posttraumatic stress, and major depression disorders have been linked to the hyper-activation of HPA axis and are directly correlated to a dysregulation in the CRF system^11,13^ and AD^5,14,15^.

Chronic stress-dependent disorders can trigger and/or facilitate AD. This effect is specific to the onset of depression late in adulthood, but not for early-age depression^65,66^. However, stress-dependent disorders such as depression, anxiety disorders, and mood disorders are often concurrent with AD. Patients with early AD display alterations in mood before or concurrently with initial signs of minor cognitive impairment or memory loss^67,68^. Recently, a unique transgenic rat model of AD, TgF344-AD (APP (Swe), (PS1ΔE9)), has been used to investigate emotional components of AD^69^. Young TgF344-AD rats were tested for spatial memory functions in a Morris water maze and for anxiety-like behavior in an elevated plus maze. TgF344-AD rats showed increased anxiety-like behavior without a significant decline in spatial memory. These results indicate that enhanced anxiety-like behavior correlates with an early stage of AD and can be used as an early marker^58^. Furthermore, depression-like symptoms can accelerate the loss of brain tissue shown in magnetic resonance imagingstudies in patients with early stages of AD^70–72^. To summarize, stress can lead to AD and accelerate neurodegeneration, but disease progression can in turn cause disruption of stress response circuits, which can exacerbate stress-like symptoms, forming a vicious cycle^66^. On the other hand, AD is caused by a combination of different factors and not only linked to high and/or chronic stress levels.

## The link between CRF and AD

As discussed above, the effect of chronic or high levels of stress leads to the hyperactivation of the HPA axis. Besides the elevation of glucocorticoids^73^, clinical studies have documented the CRF dysregulation in patients with AD^74–76^. Significant reduction of the CRF immunoreactivity (CRF-IR) was observed in postmortem tissue in the cerebrocortical areas and in the cerebrospinal fluid of patients with AD compared with that in control tissue^75,77,78^. Together with the decrease in CRF-IR, increase in the concentration of the CRF receptors (CRF-Rs) in the cortex has been directly linked to AD^74,79^. CRF-expressing neurons in the paraventricular nucleus of the hypothalamus show elevated levels of CRF mRNA in postmortem brain from AD patients compared with age-matched healthy controls^48^; however, the number of CRF-expressing cells is not altered. Calcium flux measurements and neuronal death assays in culture suggested a neuroprotective role for CRF in response to Aβ toxicity in AD brain, although this hypothesis was never confirmed by in vivo studies^80^. It has been hypothesized that CRF might stimulate non-amyloidogenic APP cleavage, exerting a beneficial effect on Aβ accumulation^81^. In vivo animal studies identified CRF as a factor that enhances cognition and memory. Upon injection in the amygdala and the ventricles of the rat brain, CRF dose-dependently improved retention of the inhibitory avoidance response and improved acquisition of a visual discrimination task^22,82^. In addition, blockage of CRF in the basolateral amygdala in rats and restoring free levels of CRF afterwards showed improved memory^83^. Notably, these are all aspects of memory and cognition that are significantly impacted in AD patients^84^.

In contrast to these findings, multiple studies have demonstrated that exposure to stress, along with elevated levels of CRF, provoked Aβ neuropathology^85–87^. For example, in the 3 × Tg-AD mice^88^, enhanced CRF signaling associated with short-term life stress in adults enhanced the progression of Aβ neuropathology^58^. In parallel, infusion of CRF directly into the hippocampus increased the concentration of two major isoforms of Aβ, namely Aβ40 and Aβ42^85,86^, therefore facilitating AD. Park et al.^89^ reported a link between this Aβ-enhancing potential of stress-induced CRF and γ-secretase activity as the underlying mechanism. Further, reduced CRF signaling in CRF-R1-deficient mice or treatment with the antagonist NBI-30775 (also known as R121919) both alleviated the stress-induced aggravation in Aβ pathology in a stress-like paradigm in AD mouse models^90,91^. In addition to increasing Aβ concentration, CRF can also enhance Tau pathology^56,92^. Enhancement of Tau phosphorylation at specific epitopes was observed in CRF-overexpressing mice and could be reversed by the specific CRF-R1 antagonist NBI-30775^93^.

To summarize, chronic stress in combination with high CRF levels can cause stress-dependent disorders, increase the risk of AD, and can lead to other diseases^94^. On the other hand, in neurodegenerative diseases, low CRF concentrations are observed^78,95,96^. Chronic stress leads to HPA axis overstimulation, followed by the generation of high CRF and GRs levels in the blood, which can cause detrimental effects to neurons and their connections. These detrimental effects can lower CRF levels in the brain leading to a decrease in its neuromodulatory functions in memory and cognition^6,58,97^.

As of today, AD remains incurable. There is a clear potential to investigate the link between CRF and AD with a focus on developing CRF-centric therapeutic approaches. Targeting CRF signaling, particularly CRF receptors, in stress disorders has been considered as a valid approach for a considerable time^10^.

## Signaling and pharmacology of CRF receptors

CRF-Rs belong to the family of G-protein-coupled receptors (GPCRs). These GPCRs mainly activate Gs, which will in turn increase adenylcyclase and stimulate enzymatic activity. Activation of the CRF-Rs, by CRF or by their other ligands, follows a two-step mechanism. First, the C-terminus of CRF binds to the N-terminus of the CRF-Rs. This first interaction causes a conformational change in the CRF-R, which stabilizes the CRF–CRF-Rs interaction, whereupon the interaction with the N-terminus of CRF initiates, and thereby triggers, cellular signaling by activating G-protein signaling^98–102^. Different ligands cause different conformational changes and consequently lead to binding of different G-proteins that will each activate distinct signaling pathways^103^. For example, in primary cells of pregnant myometrium, upon binding of UCN I with CRF-R1, there is an activation of mitogen-activated protein, which stimulates G_q_ for activation of the IP3-protein kinase signaling pathway; however, CRF binding does not activate the same signaling cascade^104^. CRF-R1 peptide agonists, such as astressin (Ki = 0,7), lack the N-terminus of the CRF (Table 1) but bind with high affinity to the extracellular binding domain to prevent binding of a CRF-R agonist. The lack of the N-terminus prevents G-protein interactions and thus inhibits downstream signaling^101,105^. Conversely, ligands such as α-helical CRF fragment 9–41 that lack the C-terminus (Table 1) can bind with low affinity (Ki = 19) and still will be able to activate the receptor^106,107^. Non-peptide antagonists or small molecule agonists, such as NBI-30775, antalarmin, and CRA-0450, bind almost exclusively to the juxtamembrane region, which partially inhibits CRF-R1 ligand binding by blocking the interaction of a ligand with the juxtamembrane domain, but cannot prevent the binding between the ligand and the extracellular domain of the receptor. NBI-30775, antalarmin, and CRA-0450 are allosteric inhibitors; they do not bind to the N-terminus to activate the receptor, but instead bind to the J-domain where they will cause a conformational change, leading to the inactivation of the receptor (Table 1).

To date, there are no specific non-peptide or small-molecule antagonists for CRF-R2^10,108^. CRF-R1 antagonists such as NBI-30775, pexacerfont, and CP-316,311 were the first allosteric GPCR inhibitors in clinical trials against depression and anxiety disorders. However, none of these compounds showed efficacy in advanced trials^109–111^. More recent pre-clinical discoveries present a more detailed view on the interaction between a ligand and the CRF-Rs. The observation of receptor association and dissociation revealed a significant difference between lead compounds. These differences could explain the disappointing outcome of previous clinical trials. Future trials could be improved by focusing on maximizing the receptor residence time or adjusting the dosage of antagonist to ensure CRF-R1 in the brain will remain occupied over longer periods^112^. In addition, novel technologies, such as positron emission tomography, genetic screening, and local viral CRF overexpression tools open up new opportunities to develop new compounds that will be more effective to influence CRF-R1 downstream signaling^10,113^.

## CRF-BP and treatment of AD

A growing body of evidence suggests a prominent role of CRF-BP in the CRF system. CRF-BP regulates CRF bioavailability in the HPA axis and can restore normal levels of CRF. It is therefore tempting to consider targeting CRF-BP as a potential therapeutic route to control CRF levels, which are disrupted in AD, to improve memory and to delay AD progression.

CRF-BP is highly conserved in gene structure throughout the evolution from invertebrates to humans^114^. Studies have indicated its importance in the regulation of CRF activity^39^. CRF-BP expression is increased in the pituitary and the brain, predominantly by stress^115^. This increased expression of the CRF-BP, specifically in the HPA axis, results in lower levels of free, i.e., CRF-BP-unbound, CRF and suppresses CRF-R1 activation and downstream signaling. CRF-BP is expressed in a highly tissue-specific pattern and differs between species. Human CRF-BP is detected in the plasma, placenta, synovial fluid, amniotic fluid, liver, the pituitary, and the brain^116^. In other species, notably in rodents, CRF-BP expression is exclusively restricted to the brain and the pituitary^32,39^. These species-specific expression patterns represent an important limiting factor in behavioral and in vivo studies for evaluating the full physiological functions of the protein in rodent stress models. In the past, three different mouse models with altered CRF-BP expression were created: two transgenic mouse lines that overexpress CRF-BP and one CRF-BP knockout model^117–120^ (Table 2). Phenotypical studies showed increased anxiety in mice with a CRF-BP-null mutation^117,121^. Unfortunately, no study has so far shown changes in memory consolidation or cognition in these transgenic CRF-BP models. In the human brain, a large portion of CRF appears to be complexed with CRF-BP and therefore likely unavailable for activating CRF-Rs and downstream events^122^. The effective concentration of free CRF is not directly related to total CRF abundance. Instead, the effective concentration is a function of both CRF and CRF-BP levels^116^. Several clinical trials that investigated the inhibition of CRF by blocking its receptor CRF-R1 as a potential treatment for CRF-dependent disorders had negative outcomes, as discussed above. The clinical studies either demonstrated multiple side effects, such as in the case of compound R121919, which caused elevated liver enzymes, or reported a complete lack of efficacy, such as in the case of CP-316,311, verucerfont, and pexacerfont^10,123^. Accordingly, such drugs that directly target the binding of CRF-BP to CRF and indirectly inactivate CRF-R1 may represent attractive next-generation candidates for the treatment of stress-dependent disorders, potentially including AD.

**Table 2:** An overview of studies to observe the role of CRF-BP by genetic or non-genetic manipulations in functional animal models

		Model	Molecular changes	References
**Genetic modifications**	*Overexpression models*	Overexpression of CRF-BP in the pituitary	Compensatory increase of CRF and arginine vasopressin in the paraventricular nucleus	^137^
			**Phenotype**	
			Increased locomotor activity and a non-significant decrease in anxiety	
		Overexpression of CRF-BP in the brain, the pituitary, and peripheral tissue	Attenuation of ACTH secretion after lipopolysaccharide treatment	^119^
			**Phenotype**	
			Increased body weight	
	*Knockout models*	CRF-BP-null mutation (CRF-BP KO)	Levels of corticosterone and ACTH were unchanged	^117, 121^
			**Phenotype**	
			Reduced body weight (male mice), increased anxiety (sex dependent)	
			Impairment of maternal aggression but no change of inter male aggression	
**Non-genetic modifications**		Acute stress condition and/or removal from adrenal glands in rats (adrenalectomy)	The acute stress condition: increased CRF-BP expression Adrenalectomy condition: decreased CRF-BP expression	^138^
		Induced stress by food deprivation in rats	Increased CRF-BP in basolateral amygdala. Decreased CRF-BP expression in the pituitary and elevated plasma corticosterone	^139^
		The effect of acute and chronic restraint stress, specific on basolateral amygdala and dorsal hippocampus in rats	Acute stress condition: increased CRF-BP mRNA expression. Chronic stress condition: no change in CRF-BP mRNA levels in basolateral amygdala. CRF-BP mRNA levels in the hippocampus were unaltered	^140^
		The effect of acute restraint stress in basolateral and central amygdala in rats	Increased expression in the basolateral amygdala. No change in CFR-BP expression in the central amygdala. No changes in CRF or CRF-Rs mRNA in both regions	^141^
		Social defeat in male rats	There may be an increase in free available CRF	^142^
			**Phenotype**	
			Impairment of social approach behavior. After adding CRF_6-33_ into the bed nuclei of the stria terminals, restored social approach	

Using the CRF-BP antagonist ligand CRF_6-33_, Behan et al.^124^ showed a restoration of normal brain levels of CRF in AD models. The application of CRF_6-33_ increased free levels of CRF and demonstrated cognition-enhancement properties in the Morris water maze paradigm of learning and memory in AD mice. This indirect approach to activate CRF-Rs does not require CRF-R agonists and is consequently unlikely to result in no characteristic side effects of CRF-R agonisms, such as disturbance in anxiety and arousal, which are characteristic to CRF-R1 and not CRF-R2^124,125^. In AD, there is no change in CRF-BP, so the total amount of hCRF does provide a readout of free levels of CRF. Therefore, releasing CRF from its binding protein would elevate the levels of available CRF in the aging brain. Consequently, CRF-BP ligands could potentially be sufficient to alleviate some of the symptoms and memory deficits in AD patients. In addition, the critical role of astrocytes in AD and other neurodegenerative diseases such as amyotrophic lateral sclerosis and multiple sclerosis is currently emerging^126,127^. Astrocytes express and release CRF-BP, and display the highest amount of membrane-bound CRF-BP. The astrocyte-associated CRF-BP reservoir might potentially play a causative role in AD and represent a therapeutic target. Astrocytes have attracted attention due to their role in disease^127^ and until now their roles as an important source of CRF-BP have not been broadly investigated in stress-related diseases or neurodegenerative disorders.

## Conclusions

Stress and AD are strongly linked to each other. Ligands and receptors of the CRF system have been investigated as therapeutic targets in neuropsychiatric disorders in numerous studies and clinical trials. Interpretation of these data is not always straightforward. As discussed above, effects of CRF as neuropeptide are highly variable depending on the location, dose, and exposure time. Further, a clear distinction must be made between functions as hormone and as neuropeptide, particularly with regard to local vs. systemic and primary vs. secondary effects. Here we provide a rationale for targeting CRF-BP as an alternative approach in AD. Admittedly, there is still a shortage of mechanistic data about CRF-BP and its possible function and distribution in the brain. Two studies investigating the potential roles of CRF-BP in AD focused on its inactivating impact/effect on CRF and UCN I interactions with their receptors^124,125^. In an animal model, by targeting the CRF-BP it was possible to achieve restoration of memory and learning the brain functions impaired in AD^125^. These findings could lead to new treatment possibilities in AD by using CRF-BP as a therapeutic target. The aforementioned studies did not investigate any other possible roles of the binding protein, its possible brain region-specific functions, or its cellular and subcellular localization^124,125^. Furthermore, one needs to consider the difference in AD prevalence between male and female subjects, and the fact that females are more susceptible to develop stress-dependent disorders, correlating with and potentially due to the different distribution pattern of CRF-BP in the brain^42,128^. Nevertheless, CRF-BP appears to be an attractive target for symptomatic treatment in AD. Inhibition of CRF-BP has the potential to boost free CRF levels, which are decreased in AD. Unlike CRF-R1 antagonists, targeting CRF-BP is unlikely to interfere with the HPA axis and to cause pertinent side effects. Clearly, more fundamental research is required prior to any progression to the clinic.
